# Label-Free Single Protein Dynamics Revealed by Metasurface-Enhanced Raman Spectroscopy

**DOI:** 10.1021/acsnano.6c04055

**Published:** 2026-07-13

**Authors:** MohammadReza Aghdaee, Sharif Zaidouni, Yeganeh Bahiraie, Anupa Kumari, Oluwafemi S. Ojambati

**Affiliations:** Department of Applied Nanophotonics, Faculty of Science and Technology, MESA+ Institute for Nanotechnology, 3230 University of Twente, Enschede 7522NB, The Netherlands

**Keywords:** energy landscape, single protein dynamics, label-free biosensing, plasmonic metasurface, Raman spectroscopy

## Abstract

A major challenge in biomedical science is a direct observation of conformational dynamics in single proteins, interacting with drug molecules, ligands, and other biomolecules. This limitation prevents access to free-energy landscapes that are fundamental to drug binding and transport and regulation of protein functions. Existing single-molecule experimental techniques rely on labels or tethering that can perturb the free-energy landscape or lack structural resolution. Here, we present an engineered plasmonic metasurface platform that enables label-free mapping of the conformational free-energy landscapes of individual proteins using metasurface-enhanced Raman spectroscopy. With single-molecule sensitivity, we resolve the predominant secondary structures adopted by bovine serum albumin protein while interacting with various drug-like functional groups in different pH conditions. We construct the free-energy landscapes and the transition pathways that reveal interconversion probabilities between α helix and either β sheet or random coil. These results reveal insights into the cooperative role of electrostatic interactions and chemical functionality on the conformational energy landscape. Our approach establishes a platform to study protein function, drug screening and testing, and protein–ligand interactions.

## Graphical abstract

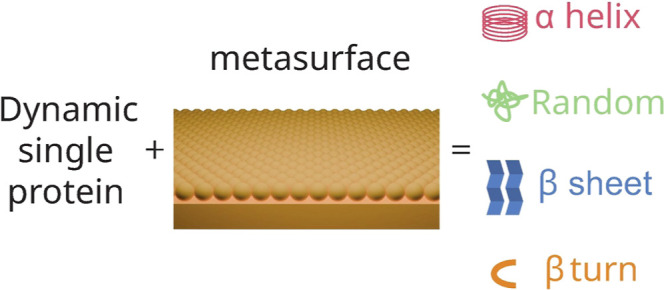

Proteins dynamically interconvert between multiple structural conformations that are sampled across a free-energy landscape, and this dynamic behavior controls protein functions and molecular interactions in a physiological environment.^1^ Particularly, protein–drug interaction is conformation-dependent, with the free-energy landscape controlling both affinity and molecular recognition.^2^ Protein conformational landscapes also influence folding pathways by defining access to transient intermediate states, which shape dynamic trajectories and interaction outcomes. Disruption of these processes leads to protein misfolding and aggregation, which are implicated in numerous diseases.^3^ Despite the central role in biomedical sciences, protein conformational dynamics is still challenging to observe directly at the single-molecule level, limiting mechanistic insights that are crucial for therapeutics,^4^ drug testing and delivery,^5,6^ and biosensing.^7,8^

Accessing protein dynamics and conformational landscapes experimentally requires an engineered approach that combines single-molecule sensitivity with protein structural resolution while also allowing unrestricted dynamics without labeling or tethering the protein. Existing experimental techniques do not simultaneously meet these important requirements. For example, conventional bulk techniques (e.g., circular dichroism^9^ and infrared spectroscopy^10^) average over vast molecular populations, thereby obscuring transient conformations that occur in dynamic situations.

Recent advances in single-molecule techniques demonstrated the ability to go beyond the ensemble averages and instead monitor real-time conformational dynamics of single proteins. However, these experimental techniques achieve single-molecule sensitivity at the cost of perturbing protein dynamics or sacrificing structural resolution. Atomic force microscopy and optical tweezers require physical immobilization of the protein to a surface or handle.^11–13^ Interferometric scattering microscopy resolves translational and rotational motion, but extracting detailed structural information is challenging.^14,15^ Single-molecule Förster resonance energy transfer (smFRET) can offer nanometer-scale spatial resolution and high temporal sensitivity for tracking intramolecular distance changes.^16–20^ However, smFRET typically requires fluorescent labels that alter protein structure and dynamics, and it is generally less sensitive to subtle secondary structure changes.^21^

Surface-enhanced Raman spectroscopy (SERS) can also achieve single-molecule sensitivity,^22^ is label-free,^23^ and provides molecular information about the protein secondary structure.^24,25^ However, the fundamental challenge of SERS is that a protein needs to be immobilized near regions of high electromagnetic field enhancements, known as “plasmonic hotspots”. The plasmonic hotspots enable pronounced SERS intensity due to (coupled) localized surface plasmons that are excited in sub-5 nm gap regions or at sharp metallic tips.^22^ Observing SERS from freely diffusing proteins requires enhanced fields in an open space to avoid constraining protein motion.^22,26,27^ Recent experiments have shown the potential of SERS-based plasmonic nanopores for peptide sequencing and nanoscale structural readout in real time.^28,29^ Other nanopore approaches have also demonstrated high sensitivity in ionic transport and transmission-based measurements.^30,31^ However, the confined space inside the plasmonic nanopore restricts protein motion, complicating measurements with large or complex protein systems.

An important but unexplored application of unperturbed protein SERS is a direct observation of protein–drug interactions under native, solution-phase conditions. For example, serum albumin, the dominant carrier protein in blood plasma, is involved in transporting diverse endogenous ligands and drugs. Molecular binding can shift conformational equilibria and modify secondary structures, with direct consequences for binding kinetics and off-target interactions.^32^ Therefore, it is highly scientifically and technologically relevant to experimentally observe albumin interactions with drug molecules. Such an observation can provide direct insight into the binding kinetics and efficacy of drugs, which are important in medicine and pharmaceutical science.

In this work, we present a label-free plasmonic metasurface platform to probe protein interactions with drug-like molecules via Raman spectroscopy. Our approach achieves single-molecule sensitivity and unperturbed protein dynamics by using a plasmonic metasurface with open-space hotspots that illuminate proteins in solution. We resolve the secondary structures and dynamics of bovine serum albumin by tracking changes in the amide-I vibrational band. The population and evolution of the secondary structures enable the construction of the conformational landscape and transition pathways, which are strongly modified by the interactions of the protein with different drug molecules. Our results show that electrostatic interactions primarily determine the conformational landscape and transition pathways.

## Results and Discussion

### Plasmonic Metasurface

Our experimental approach is based on a plasmonic metasurface, which is made of a closely packed monolayer (0.8 nm interparticle spacing) of Au nanoparticles (80 nm diameter) on a Au film (Figure 1a; see Methods for sample fabrication). The dominant scattering resonance of the fabricated structure falls into the near-infrared region (Figure 1b). The measured averaged dark-field spectrum has a broad and dominant resonance at 900 nm, which is 20 nm blue-shifted from the boundary element method (BEM) simulation (see the Methods). We attribute this 20 nm shift and the slightly broader resonance of the experimental data to nanoparticle size dispersion and interparticle spacing variation (see the Methods). Overall, the experimental dark-field spectrum is in agreement with the simulation results.

**Figure 1:**
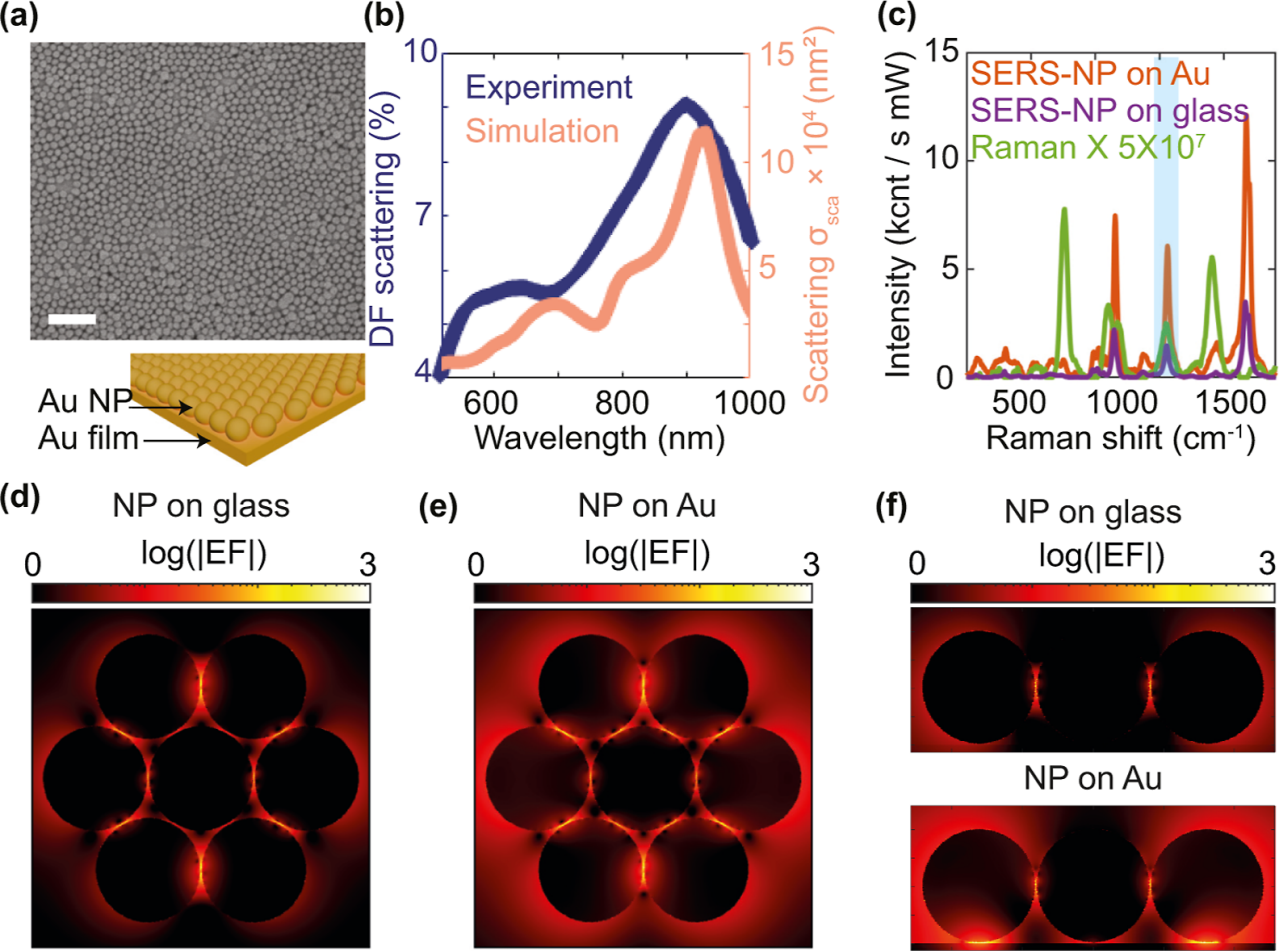
Plasmonic metasurface for protein-dynamics measurements. (a) Scanning electron microscope image of the fabricated plasmonic metasurface (scale bar: 250 nm). Inset: schematic of the metasurface, consisting of a closely packed monolayer of Au nanoparticles on a Au film. (b) Experimental dark-field scattering spectrum and numerically calculated scattering cross section. (c) SERS spectra of diffusing biphenyl-4-thiol (BPT) in solution measured on a Au-nanoparticle monolayer on a Au film (orange) and on a glass film (purple), compared with the conventional Raman spectrum of BPT in solution (green). The BPT concentration is 8.5 μM for SERS and 8.5 mM for Raman. The highlighted area shows the Raman peak at 1280 cm^–1^ used for the SERS enhancement-factor calculation. (d,e) Simulated electric-field enhancement, |EF|, in the horizontal plane at the nanoparticle-axis height for a Au nanoparticle monolayer on glass (d) and on Au film (e). (f) Corresponding |EF| distributions in the vertical plane for the Au nanoparticle monolayer on glass film and on Au film. Field maps are calculated at the respective scattering-peak wavelengths.

To quantify the SERS enhancement, we compare the Raman and SERS intensities of diffusing biphenyl-4-thiol (BPT) as only a Raman reporter on two samples: (1) a Au nanoparticle monolayer on a glass film and (2) the same monolayer on a Au film. We use the standard concentration-normalized enhancement factor relation, $EF=\left(\frac{I_{SERS}}{I_{Raman}}\right)\left(\frac{C_{Raman}}{C_{SERS}}\right)$ , where *I*_SERS_ and *I*_Raman_ are the measured intensities of the Raman peak and *C*_SERS_ and *C*_Raman_ are the corresponding BPT concentrations used in the SERS and Raman measurements, respectively. We obtain the SERS enhancement factors of EF ≈ (3 ± 0.4) × 10^7^ on the nanoparticle on glass substrate and ≈(1 ± 0.2) × 10^8^ on the nanoparticle on Au substrate obtained from the Raman peak at 1280 cm^–1^ (Figure 1c). The spectral shift between the Raman and SERS peaks is due to interactions with the metallic surface that alter the molecular environment through chemical binding and charge-transfer effects, shifting vibrational frequencies.^33^ However, the observed SERS spectrum is consistent with the literature.^34^

The electromagnetic field near the SERS substrate is strongly enhanced due to the interactions between the Au nanoparticles and the underlying Au film (Figure 1d–f; see Supporting Information. A) Localized surface plasmons from each nanoparticle couple with both neighboring particles and their image charges in the Au film. These multiple plasmonic couplings result in a strong localization of induced surface charges and steep potential gradients at the nanogaps. The strongly enhanced fields, therefore, give rise to an enhanced Raman intensity from target molecules diffusing above the metasurface. Simulations show a maximum field enhancement of 450 for the nanoparticle on the Au substrate and 360 for the nanoparticle on the glass substrate. The glass-based substrate exhibits weaker field enhancement across different vertical *z* positions (see discussion in the Supporting Information. A) Both simulations and experiments confirm that coupling between the nanoparticles and the Au film yields a stronger field enhancement. Consequently, we employ Au nanoparticles on a Au film in our further measurements.

### Label-Free Single-Protein Dynamics

We employ the plasmonic metasurface to probe the dynamics of single molecules of BSA protein diffusing near the illuminated metasurface (for estimation of the number of molecules, see Supporting Information. B). While such closely packed Au nanoparticle-on-film metasurfaces have previously been explored for SERS enhancement,^22,26^ here we use this open plasmonic architecture to directly observe the conformational dynamics of diffusing single proteins in a label-free manner without immobilization or molecular confinement. To experimentally access the protein–surface interactions, a focused laser beam with a power of 300 μW at 640 nm illuminates the metasurface (Figure 2a) through a 1.4 NA oil immersion objective (see Supporting Information. C). With a 20 nM protein aqueous solution on the metasurface, we acquired Raman spectra every 100 ms for 2 min on at least 50 different positions on the sample. Our analysis focuses on the amide I region (1640 cm^–1^–1680 cm^–1^) of the Raman spectra to extract the secondary structure of BSA. The amide I band mainly originates from the C=O stretching vibration of the peptide backbone. The Raman peak position is highly sensitive to the protein secondary structure, indicating the presence of α helices (1650 cm^–1^–1660 cm^–1^), β sheets (1660 cm^–1^–1670 cm^–1^), β turns (1670 cm^–1^–1680 cm^–1^), or random coil structures (1640 cm^–1^–1650 cm^–1^) across the amide I region (see Supporting Information. D). We note that our measurement is mainly sensitive to the dominant secondary structure in the protein, resulting in a single Raman peak, unlike in the bulk Raman measurements where multiple Gaussian functions fit the amide I peak due to averages over multiple conformations.

**Figure 2:**
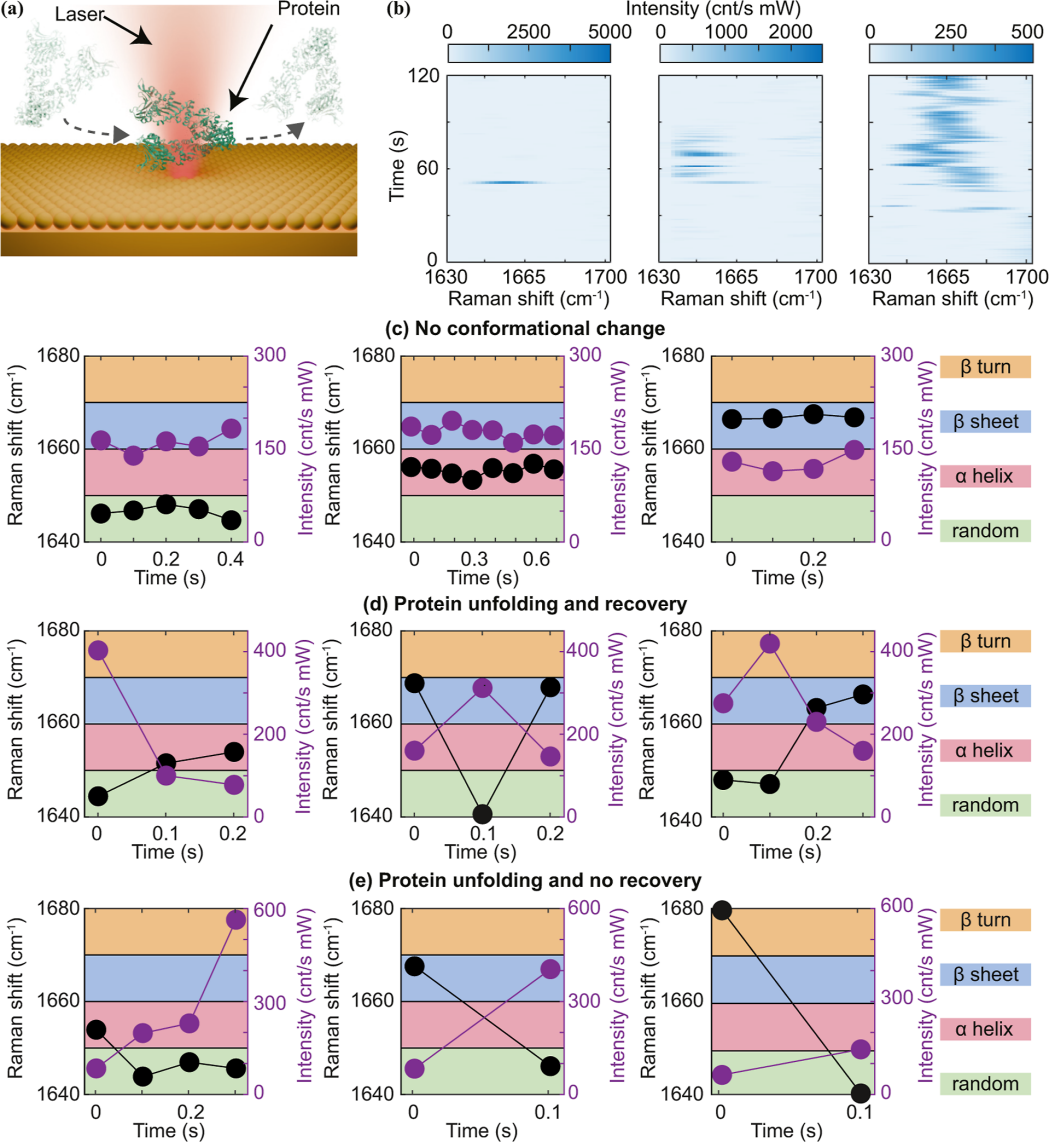
Dynamics of a single protein diffusing on the plasmonic metasurface. (a) Schematic of the experimental setup. A 640 nm laser with a focused power of 300 μW illuminates the metasurface, probing diffusing single-molecule protein molecules.^35^ (b) Time-resolved Raman maps of the amide I band acquired show diverse interaction events: minimal interaction (left), transient multimolecule events (middle), and prolonged single-molecule interactions (right). (c–e) Raman shift (black) and intensity (purple) in the amide I region vs time show three interaction regimes: (c) no conformational change; stable Raman intensity and peak position indicate preserved secondary structure. (d) Conformational changes; time-dependent spectral shifts indicate (reversible) structural transitions. (e) Protein unfolds and no recovery; rapid redshift and intensity decay indicate unfolding. Structural conformations are color-coded: random (green), α helix (pink), β sheet (blue), and β turn (orange).

The diffusing single proteins occasionally approach the metasurface within the illuminated area, producing a detectable Raman signal. Single diffusing protein events range from no or single-molecule interactions (63% of the total measured spectra; Figure 2b left, and Supporting Information. E), to multiple short-lived interactions (27%, Figure 2b center), and long-duration events lasting several seconds (10%, Figure 2b right). Based on our data, we identify three distinct interaction scenarios between the protein and the metasurface. In the first scenario, the protein remains within the illuminated volume with a fairly constant Raman intensity and peak position, indicating a minimal fluctuation of the vertical position, and the protein retains its conformation (Figure 2c). In the second scenario, the protein undergoes conformational changes: it unfolds, transits through the disordered random coil state, and then recovers to another secondary structure (Figure 2d). In the third scenario, there is a rapid unfolding—loss of α helical, β sheet, or β turn content—as seen by a redshift in the amide I band (Figure 2e). In the following sections, we demonstrate the possibility of modifying these observed protein dynamics via pH and functional groups.

### pH-Dependent Protein Conformation

We analyze the distribution of the observed Raman peaks of the single proteins at different pHs (for more details, see Supporting Information. F). The Raman peak histograms show a broad distribution of Raman shifts across the amide I region, indicating the presence of multiple secondary structures (Supporting Information. G). To resolve the spectral diversity, we apply principal component analysis (PCA), focusing on the first two principal components: PC1 and PC2 (Figure 3a–c). PCA effectively clusters the data into four distinct regions corresponding to different secondary structures: α helix, β sheet, β turn, and random coil. Furthermore, we quantify the fractional contribution of each secondary structure element by classifying the individual events (Figure 3d–f).

**Figure 3:**
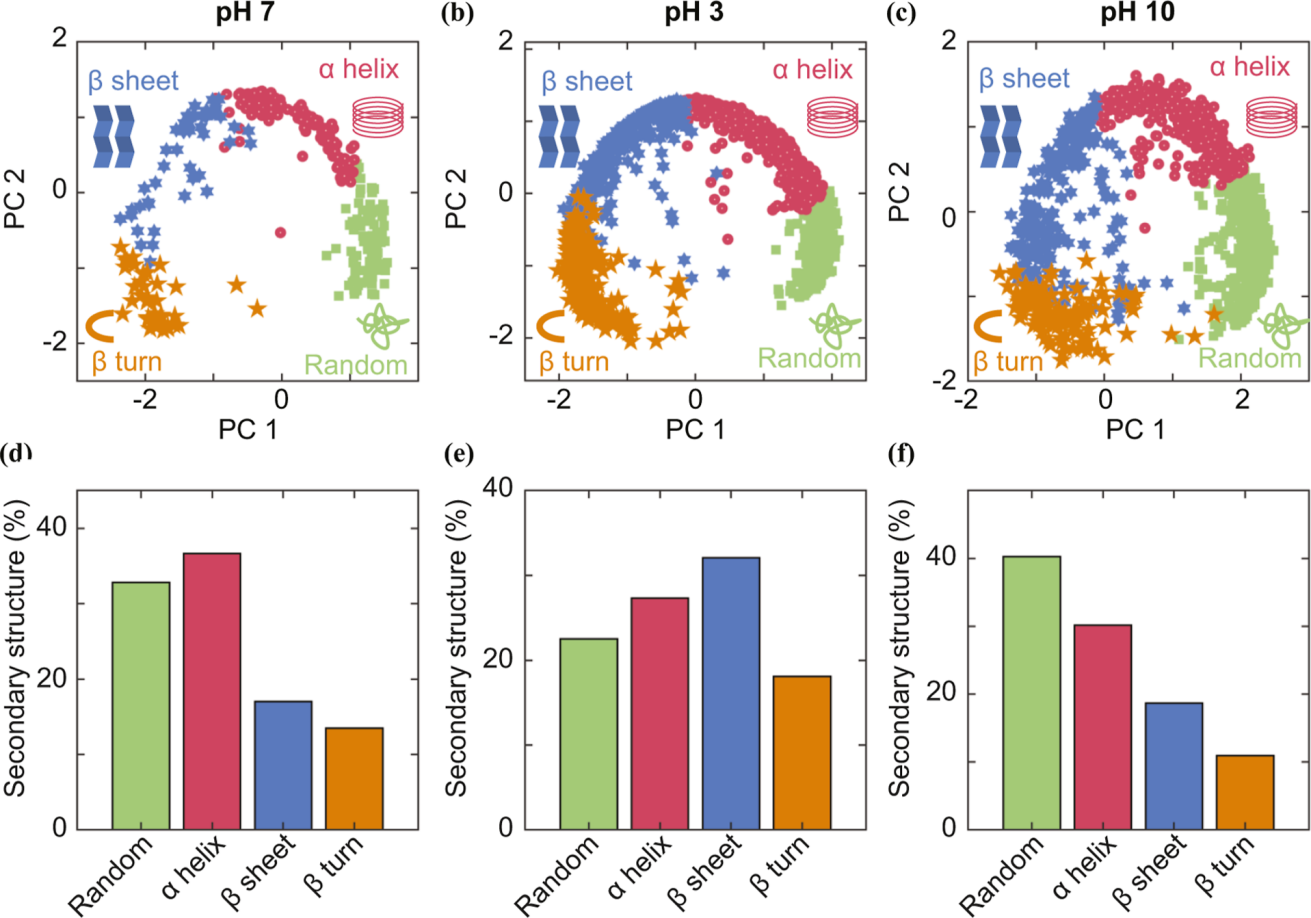
Single-molecule Raman spectroscopy analysis of the BSA secondary structure. (a–c) Principal component analysis (PCA) of individual Raman spectra, revealing clustering associated with α helix, β sheet, β turn, and random coil structures at pH 3, 7, and 10; the insets show representative protein conformations corresponding to the identified secondary structures. (d–f) Quantification of secondary structure populations based on spectral classification at pH 3, 7, and 10.

At pH 7, the α helical structure is the dominant secondary conformation, contributing 37% of the total measured spectra, followed by random coil (33%), while β sheet and β turn structures contribute 17% and 13%, respectively. To a large extent, the observed conformational distribution agrees with bulk measurements reported in the literature, where the α helix is the dominant conformation (46%), followed by random coil and turn structures (40%), and β sheet components (18%).^36–39^

Our observations show that extreme pH conditions significantly alter the secondary structure of a single diffusing protein (Figure 3e,f and summary in Figure 4a). There is an acid-induced structural change and base-induced destabilization of the native α helical conformation. In the acidic condition, the protein adopts a predominantly β sheet conformation, driven by an increased protonation of aspartic acid and glutamic acid side chains. These negatively charged side chains generate electrostatic repulsion at neutral pH, whereas protonation under acidic conditions reduces this repulsion and thereby enhances protein aggregation. This protonation promotes inter- and intramolecular interactions that stabilize β sheet conformation.^40,41^ Under basic conditions, the protein predominantly adopts disordered conformations. Increased pH promotes deprotonation of amino acid side chains, leading to weakened stabilizing intramolecular interactions. As a result, the hydrogen bonding that supports the α helical structure is disrupted.^42^ Consistent with these mechanisms, our pH-dependent single-protein observation agrees with the bulk Raman measurements in the literature.^43,44^

**Figure 4:**
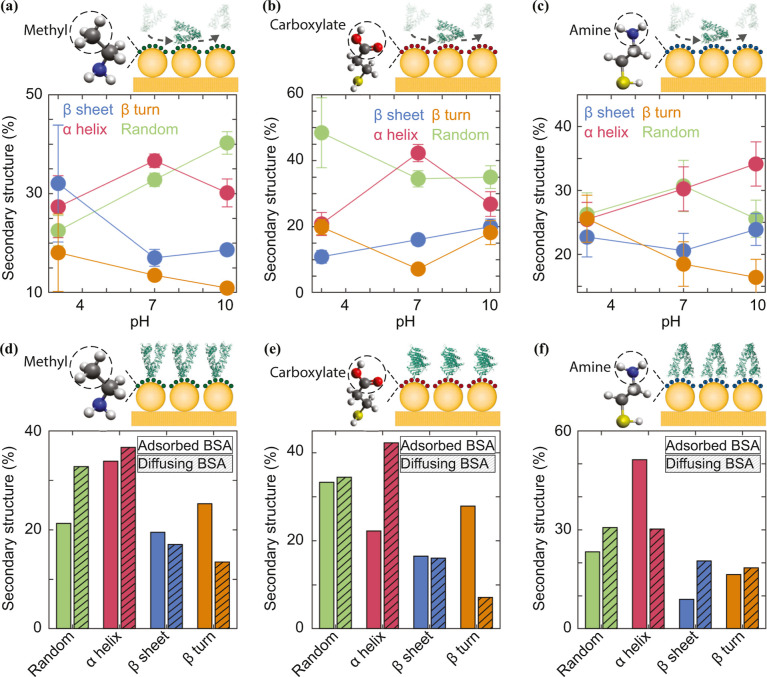
Effect of drug functional groups and pH on the BSA secondary structure. (a–c) Quantification of secondary structure populations of diffusing BSA at single-molecule level across pH 3, 7, and 10. (a) The surface of the metasurface is functionalized with ethylamine to expose a methyl functional group (neutral surface), (b) with 3-mercaptopropionic acid to expose a carboxylate functional group (negative surface), (c) with cysteamine hydrochloride with ethylamine to expose an amine functional group (positive surface). Quantification of secondary structure populations of adsorbed protein (bar) and diffusing protein (hatched bar) based on spectral classification of the secondary structure across (d) the methyl group; (e) carboxylate group; and (f) amine group. The error bars are the standard error of the mean obtained from individual time-resolved measurements.

### Protein Interactions with Drug-Relevant Functional Groups

We investigate protein-functional-group interactions, which contribute to binding behavior of the protein with molecules (e.g., in a drug). Our approach employs a reductionist but controlled model to describe drug–protein interactions to gain mechanistic insight. To connect our measurements to pharmaceutically relevant chemistry, we examine amine, carboxylate, and methyl functional groups that are present in cysteamine (a clinical drug for cystinosis^45^), 3-mercaptopropionic acid, and ethylamine, respectively. These functional groups are widespread in pharmaceuticals, for example, carboxylic acids in aspirin, ibuprofen, and naproxen and methyl groups in lidocaine, paracetamol, and diazepam. We functionalize the plasmonic metasurface with these molecules such that protein is exposed to the respective functional groups, including different charges at different pH conditions (see Supporting Information. H). The functionalization layers introduce only a subnanometer spacer between the protein and the Au surface, which remains small compared with the spatial extent of the plasmonic near-field. Therefore, although the functionalization may slightly modify the absolute SERS enhancement, the effect is systematic within each surface condition and does not affect the relative spectral comparisons since the secondary-structure analysis is based primarily on Raman peak positions rather than absolute signal intensity.

As the protein interacts with the carboxylate group (Figure 4b), the α helical content is maximal at pH 7 (42%), similar to the trend observed on the methyl group (Figure 4a). The most notable change at pH 3 is an increase in the random coil structure to 48% and a decrease of β sheet to 11%, compared to the methyl functional group, with 18% and 32%, respectively. These structural changes indicate a loss of structural order due to the protein’s net positive charge under acidic conditions, which promotes strong electrostatic attraction to the negatively charged functional group on the metasurface. As a result, the protein experiences temporary adsorption and conformational rearrangement upon contact, leading to a disruption of its native secondary structures.

Similarly, the amine functional group leads to an increased random coil content at pH 7 (31%, Figure 4c) due to the electrostatic attraction of the BSA (with a net negative charge at pH 7) to the positively charged surface. At basic pH, electrostatic attraction between the negatively charged protein and protonated amine group leads to charge regulation and screening of intramolecular repulsion, stabilizing α-helical structure despite the alkaline environment. Together, these results indicate that electrostatic interactions determine the protein structure, resulting in partial unfolding or structural rearrangements.

To disentangle surface-induced effects, we measured the secondary structure of proteins irreversibly adsorbed on the plasmonic metasurfaces after prolonged incubation of the protein solution on the metasurface followed by rinsing to remove unadsorbed proteins (Figures 4d–f, Supporting Information. I). On the metasurface with a methyl group (Figure 4d), BSA retains a dominant α helical content. The most notable effect is an increase in the β-turn content to 25%, two times higher than in the dynamic measurements. This observation aligns with previous infrared spectroscopy studies, which also reported an enhanced β turn formation upon adsorption.^46^ We also examined the β turn structure under crowded conditions by increasing the protein concentration from the single-protein regime (20 nM) to a high-concentration regime (500 μM), where repeated and overlapping interaction events promote intermolecular interactions and molecular crowding effects (Supporting Information. J). Under these conditions, we observed an increase in both the β turn and β sheet contents. This result is consistent with the results obtained for the adsorbed protein. This behavior suggests that molecular crowding promotes the formation of β structures through increased intermolecular interactions, which can trigger protein aggregation.^47^

Similarly, on the metasurface with the carboxylate group (Figure 4e), the β turn content rises more strongly (20%), with a loss of α helix, suggesting partial unfolding driven by electrostatic repulsion between the surface and negatively charged BSA domains. This effect is absent in the dynamic measurements where proteins interact only transiently with the surface. On the metasurface with the amine group (Figure 4f), BSA shows the highest α helical content (51%) and suppressed random coil and β sheet contributions, reflecting stabilizing electrostatic attraction; the resulting distribution closely matches the result in bulk solution,^36,46^ whereas in the dynamic case the reduced interaction time yields lower helicity. Together, these surface-dependent structural modifications provide an essential comparison for interpreting the dynamic SERS measurements that are more biologically relevant.

### Conformational Free-Energy Landscapes of Protein

We construct free-energy landscapes of protein–molecule interactions for four BSA secondary-structure conformations. The conformational free-energy landscape is basically a graph of free energy vs a reaction coordinate that underlies the thermodynamics of accessibility of the secondary structures. The free-energy minima correspond to the most populated and stable secondary structures. To construct the free-energy landscape, we convert the relative state occupancies of each secondary structure using the Boltzmann factor (see Supporting Information. K).

With the methyl functional group, the lowest energy secondary structure varies from β sheet, α helix, to random coil at pH 3, 7, and 10, respectively. This modification of the energy landscape reflects pH-controlled intramolecular electrostatics rather than interaction with the functional group since the methyl group is neutral. Across all pH values, the β-turn state remains the highest in free energy, corresponding to a low equilibrium population and limited conformational transitions.

At pH 7, the balance of electrostatic interactions and hydrogen bonding favors the native α-helical structure, yielding the lowest free-energy minimum, consistent with reported single-BSA energy landscapes.^48^

With the negatively charged carboxylate group (Figure 5d–f), the landscape at pH 7 and 10 closely resembles that of the methyl functional group, indicating that the protein conformation is primarily governed by solution-phase electrostatics. There is therefore minimal physical interaction with the surface due to electrostatic repulsion between BSA and the carboxylate since they are both negatively charged at pHs 7 and 10. In contrast, under acidic conditions, the protonation of the protein, combined with electrostatic attraction to the negatively charged surface, disrupts the stabilizing intramolecular contacts, destabilizing the β-sheet structure and promoting random conformation. Similarly, the amine functional group shows a distinct pH dependence arising from strong protein-functional group interactions that is primarily driven by electrostatics. For acidic and basic conditions, it appears that the positively charged amine group interacts with the negatively charged side chains, thereby modifying the solution-phase conformation, to either result in random coil or α helix at pH 3 and 7, respectively. Overall, the free-energy landscapes show that functional-group chemistry and pH play a significant role, thereby controlling conformational stability and structure.

**Figure 5:**
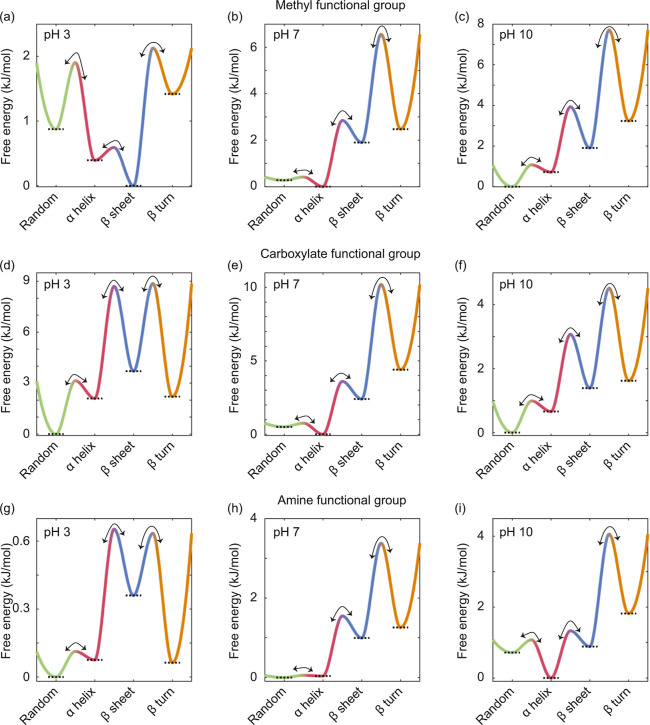
The conformational free-energy landscapes of protein under different pH and three different surface functionalizations. The free-energy landscapes show the relative stabilities of the four secondary structure conformations (random coil, α helix, β sheet, and β turn) for BSA at pH 3, 7, and 10 on metasurfaces with the methyl functional group (a–c), carboxylate functional group (d–f), and amine functional group (g–i).

### Transition Pathways and Dissociation Kinetics of Protein

We examine transitions among secondary-structure states that occur at longer time scales (>100 ms) across different pH conditions and drug-like surface functional groups. With the aid of a directed graph (Figure 6a–i), we obtain a network diagram that illustrates relative transition frequencies between different structural states, with node sizes that reflect the relative structure population, and arrow thickness that is proportional to the transition percentage.

**Figure 6:**
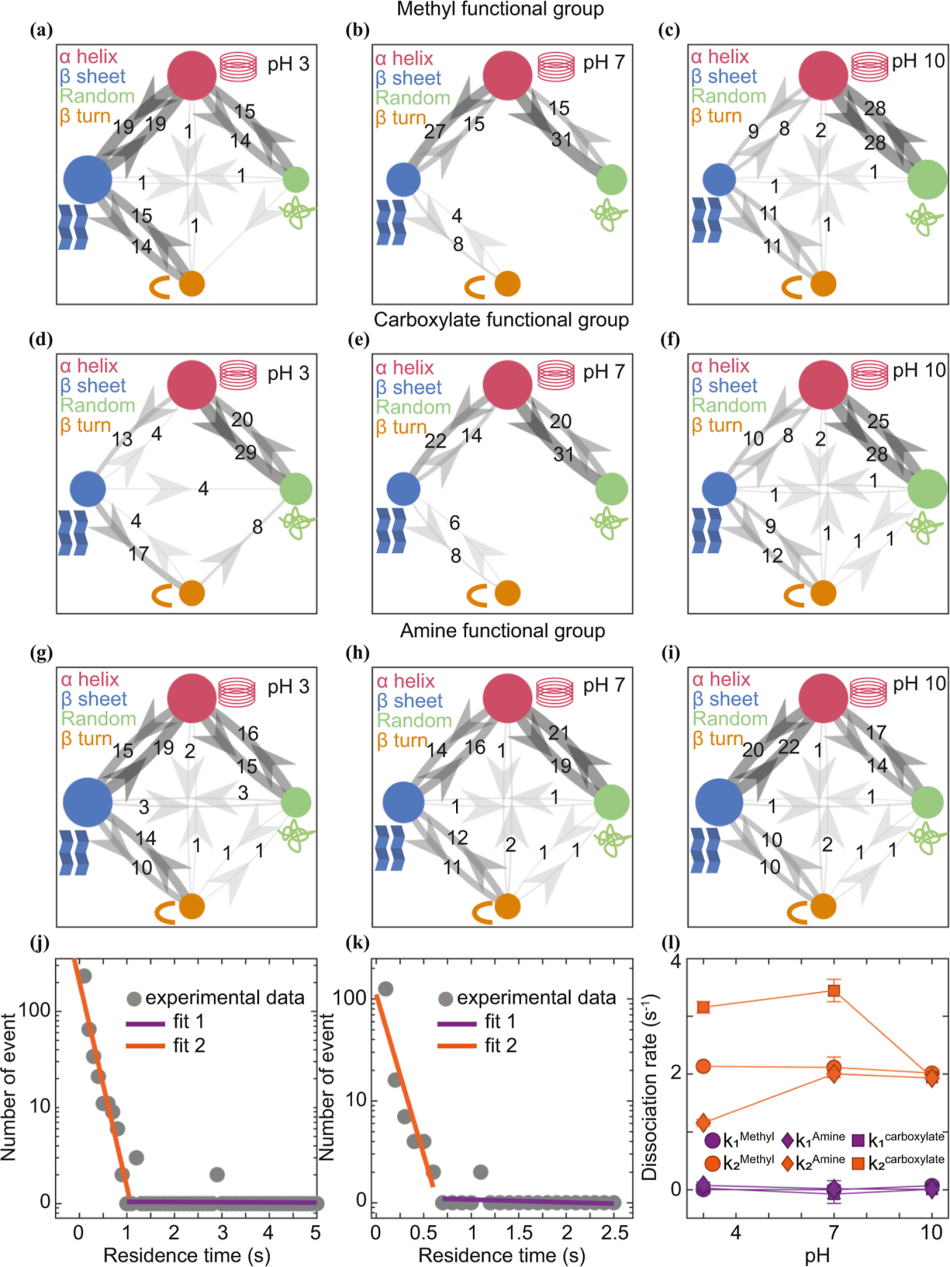
Directed graphs showing transition networks among secondary structural elements under different pH conditions on three different surface functionalizations. (a–i) Each row corresponds to a surface functionalization with methyl (a–c), carboxylate (d–f), and amine functional groups. Arrows indicate directional transitions between conformations, with associated percentages. Thicker and darker arrows represent more frequent transitions. The insets show representative protein conformations corresponding to the identified secondary structures. (j,k) Representative histograms of BSA dissociation events over time, fitted with a biexponential decay, representing fast and slow dissociation rates, respectively. The gray circles denote experimental data, while the orange and purple curves correspond to fits for the components. (j) Extracted dissociation rates (*k*_1_ and *k*_2_) for BSA on different surface functionalization across pH 4, 7, and 10.

Across all conditions, the conformational dynamics are dominated by bidirectional transitions between α helix and both β sheet and random coil, while transitions involving β turns are rare. With the methyl group, pH strongly influences the transition pattern: β sheets dominate at acidic pH, whereas at pH 7 and 10, transitions from α helix to random coil and β sheet structures and from random to α helix dominate, respectively (Figures 6a–i). The functional groups methyl and carboxylate further influence these dynamics (Figures 6d–i): with the carboxylate group, acidic conditions result in an increase in α helix to random coil transitions, while on the amine group, the transitions remain similar across pH values. In all cases, the α helix and β sheet interconversion consistently dominates the transitions.

To gain further insight into the interaction dynamics, we analyzed the dissociation kinetics of the single BSA molecules. The number of events as a function of time follows a biexponential distribution that has two distinct dissociation rates: a slow dissociation rate (*k*_1_) and a fast dissociation rate (*k*_2_, Figure 6j,k). The slower kinetic component (*k*_1_) corresponds to strong and long-lived surface interactions involving proteins adsorbed onto the surface. In contrast, the faster component (*k*_2_) reflects weaker and short-lived interactions and is associated with more dynamic or loosely bound conformations.

Kinetic analysis shows that *k*_1_ remains constant across all conditions, indicating that long-lived protein–surface interactions are insensitive to pH conditions and functional groups (Figure 6l). With the methyl group, *k*_2_ is also stable across pH values, consistent with reversible transitions dominated by nonelectrostatic interactions. In contrast, *k*_2_ strongly depends on pH on other functional groups: it is highest with the carboxylate group at pH 3 and 7 due to repulsion of the negatively charged BSA domains and lowest with the amine group at pH 3, where transitions mainly involve β sheet and α helix. These trends reflect the protonation equilibria of surface functional groups: carboxylate and amine groups (with dissociation constants of approximately p*K*_a_ ∼ 4–5 and p*K*_a_ ∼ 10, respectively) exhibit pH-dependent kinetics due to charge regulation, whereas methyl groups, lacking ionizable sites, remain pH-independent. Consequently, the p*K*_a_-defined functional group governs the strength and lifetime of short-lived protein–surface interactions captured by *k*_2_.

## Conclusion

In summary, we demonstrate a new experimental method to observe the dynamics of a diffusing protein interaction with drug-like molecules using metasurface-enhanced Raman spectroscopy. The uniqueness of our approach is based on the combination of label-free and tether-free single-protein sensitivity with secondary-structural resolution under physiological conditions. This approach enables the construction of the free-energy landscapes and conformation transition pathways involved in dynamic protein interactions. As a proof of principle, we employ a simple protein–drug interaction model by investigating the protein, BSA, interacting with functional groups in various drug molecules. Our results reveal the most stable and populated conformations across different experimental conditions from free-energy minima, as well as other less accessible states. We further observe rich interconversion dynamics between α helix and either β sheet or random coil. These dynamics are largely governed by electrostatic interactions, controlled by functional-group chemistry and pH. We anticipate future experiments to explore the modification of the energy landscape by other nanoscale forces such as hydrophobic, van der Waals, and mechanical forces. In addition, our work opens up exciting possibilities to explore faster dynamics of the secondary structure with additional improvement in the field enhancement.

The plasmonic metasurface enables new possibilities to observe label-free and unrestricted dynamics of biomacromolecules, including large proteins, ribonucleic acids, and complex carbohydrates. This platform provides a powerful tool for investigating protein folding, misfolding mechanisms, and protein–protein interactions. Furthermore, our approach enables systematic studies of protein interactions with drug molecules, toxins, and ions, providing a general route to interrogate how chemical functionalities and electrostatics reshape biomolecular structure and dynamics. A biomedical application of such interactions is in early-stage drug screening, drug testing, delivery, and transport, where conformational dynamics directly influence binding affinity, stability, and function. Moreover, our work is important at solid–liquid interfaces, where surface charge, pH, and nanoscale forces strongly influence the behavior and function of proteins. These conditions can model key physicochemical features of biomolecular interfaces such as membranes, micelles, and vesicles under physiologically relevant conditions.^49,50^ In addition, the metasurface-based system can complement and validate protein structure predictions of machine learning and computational tools such as AlphaFold, by providing experimental access to dynamic conformational structures beyond static predictions.

## Methods

### Optical Measurement

The metasurface substrate was placed in a 3D-printed chamber with a diameter of 10 mm and a height of 0.5 mm. A 20 nM protein aqueous solution was deposited onto the metasurface, and a refractive-index-matched coverslip was placed on top to minimize evaporation and optical reflections at the interfaces. We use a low 300 μW laser power to minimize photothermal effects and potential changes in protein conformation. In total, approximately 60,000 spectra were analyzed for each pH and surface charge condition. A custom-built microspectrometer was used for both SERS and dark-field measurements. For more details, see Supporting Information. C and ref 51.

### Sample Fabrication

#### Materials

In this study, we purchased the Au nanoparticles from BBI Solutions and the ethylamine, hexane, cysteamine hydrochloride, 3-mercaptopropionic acid, and acetone solutions from Sigma-Aldrich. BSA was obtained from Merck Life Science. Microcentrifuge tubes are 2.0 mL safe-Lock tubes from Eppendorf. The silicon wafer (type p/boron/100) was obtained from Okmetic.

#### Template-Stripping

We prepared the Au substrate using the template-stripping method. A 100 nm layer of Au was coated on the silicon wafer using a thermal vapor deposition with a deposition rate of 0.3 Å/s. Then, we coated the surface of the Au with a thin layer of UV epoxy (Thorlabs, NOA61) and glued round glasses (diameter 8 mm) to the surface. The glass-glued wafer was cured for 40 min under a UV light source with a peak wavelength of 365 nm. The wafer is stored at room temperature in the air, and the glass substrates glued to the Au film can be peeled off as required.

#### Au Nanoparticle Monolayer Fabrication

The fabrication process for the Au nanoparticle monolayer was as follows: the colloidal Au nanoparticles were capped with citrate. To remove the citrate from the nanoparticles, 1 mL of Au nanoparticles was mixed with 1 mL of acetone in a microcentrifuge tube. The reaction mixture was centrifuged at 5000 rpm for 10 min, and then the supernatant was removed. The remaining solution was sonicated for 1 min to redisperse the Au nanoparticles. Then, 1 mL of a 1 mM ethylamine solution was added to the microcentrifuge tube. We assemble the nanoparticle monolayer inside a 3D-printed chamber with a diameter of 25 mm and a height of 10 mm (see Supporting Information. L). The colloidal Au nanoparticles, mixed with ethylamine, are transferred to the chamber, followed by the addition of 0.5 mL of hexane. The immiscibility of the two liquid phases (hexane and water) resulted in the formation of a water/organic interface, with the water phase at the bottom.^52^ Upon the addition of 1 mL of acetone, the nanoparticles became trapped at the water/hexane interface. The formed Au nanoparticle monolayer was transferred to the template-stripped Au substrate. The fabricated Au nanoparticles on a Au film were left in a nitrogen box to dry for 24 h.

### Numerical Setup

We employ the BEM to solve the full Maxwell’s equations for plasmonic nanostructures. Details of our BEM implementation are provided in ref 51. To enable simulation of the plasmonic metasurface, we approximate the illuminated region as a cluster of seven closely packed nanoparticles.^53,54^ This simplification reflects the effective area probed in the experiment and significantly reduces computational cost.

## Supplementary Material



## Notes

Electric field distribution on the plasmonic metasurface; estimation of the number of molecules; optical setup; assignment of secondary structures from Raman peaks; transition event frequency; data analysis procedure; histogram of Raman peaks for different pHs; charge parameters for protein–surface electrostatics; secondary structure of adsorbed proteins; concentration dependence of protein secondary structure; protein conformation free-energy landscape; and fabrication of the plasmonic metasurface (PDF)
