# Supplementary material for: Label-Free Single Protein Dynamics Revealed by Metasurface-Enhanced Raman Spectroscopy
Source: ACS Nano. 2026 Jul 13;20(29):20599–610. doi: 10.1021/acsnano.6c04055 (PMC13421998; doi:10.1021/acsnano.6c04055)
Supplement: Supplementary file 1 [file nn6c04055_si_001.pdf]

Supplementary information of label-free single protein dynamics revealed by metasurface-enhanced Raman spectroscopy

MohammadReza Aghdaee, Sharif Zaidouni, Yeganeh Bahiraie, Anupa Kumari, Oluwafemi S. Ojambati*

Department of Applied Nanophotonics, Faculty of Science and Technology, MESA+ Institute for Nanotechnology, University of Twente, Enschede, 7522NB, The Netherlands..

*Corresponding author: o.s.ojambati@utwente.nl;

SI. A Electric field distribution on the plasmonic metasurface

We used the boundary element method (BEM) to calculate the plasmonic enhancement profile of the metasurface. We simulate the electric field distribution around a representative seven-nanoparticle-on-mirror configuration (Supplementary Fig. 1a). The electric field intensity in the vertical plane reveals strong confinement at both the nanoparticle–nanoparticle spacing and the gaps between the nanoparticles and the Au substrate, indicative of coupled plasmonic hotspots (Supplementary Fig. 1b). The maximum electric field intensity as a function of vertical position (z-direction) on both substrates, nanoparticle on glass substrate and nanoparticle on Au substrate, shows a rapid decay, indicating that the enhancement is strongly confined to within tens of nanometers from the nanoparticle surface (Supplementary Fig. 1c). The maximum amplitude of the electric field for the nanoparticle on Au substrate reaches 450, and the nanoparticle on glass substrate reaches 360. This strong field localization of the nanoparticle on Au substrate enables sensitive single-molecule detection within the vicinity of the metasurface. To evaluate the spatial decay of the plasmonic near field, we calculated the electric field intensity distribution (|EF|) at multiple vertical planes on the nanoparticle–film interface. The maps (Supplementary Fig. 1d) show that at Z = 0-11 nm, strong field localization occurs in the interparticle spacing, forming intense hot spots. With increasing Z, the enhancement decays; at distances beyond

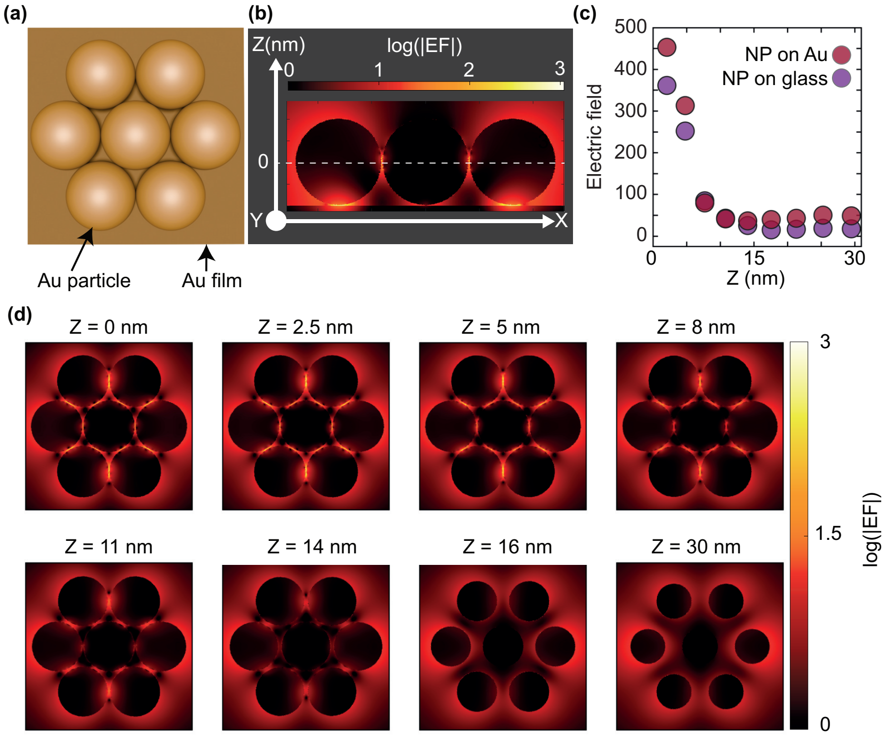

Supplementary Fig. 1 Simulated electric field distribution of the plasmonic metasurface. **(a)** Schematic of the nanoparticle arrangement used in the simulations. **(b)** Electric field distribution (|EF|) of the plasmonic metasurface in the vertical XZ-plane at $Y = 0$ nm. **(c)** Maximum electric field amplitude of nanoparticle on Au and nanoparticle on glass substrates in the XY-plane as a function of Z, with $Z = 0$ corresponding to the center of the nanoparticle array. **(d)** Electric field distribution in the XY-plane at different vertical positions ($Z = 0$–80 nm).

$\sim$14 nm, the high-field regions shrink significantly. These results confirm that the plasmonic near field is confined to the vicinity of the metasurface. To assess whether BSA molecules can access the plasmonic hotspot region between closely spaced Au nanoparticles, we considered the geometry of the nanoparticle array together with the molecular dimensions of BSA. Each Au nanoparticle (Au NP) has a diameter of 80 nm and is coated with an ethylamine ligand shell, which introduces a separation of approximately 0.8 nm between adjacent nanoparticles (Supplementary Fig. 2a). Given BSA's approximate dimensions of $4 \times 4 \times 14$ nm, it can readily fit into the interparticle gaps and approach within about 12 nm of the spacing center. This proximity is sufficient for BSA to experience the localized plasmonic near-field, enabling it to probe regions of strong enhancement (up to two orders of magnitude higher than the incident field) and thus allowing single-molecule Raman detection (Supplementary Fig. 2b).

We further analyzed the electric field distribution as a function of the excitation wavelength. The simulations show that the presence of the Au substrate is

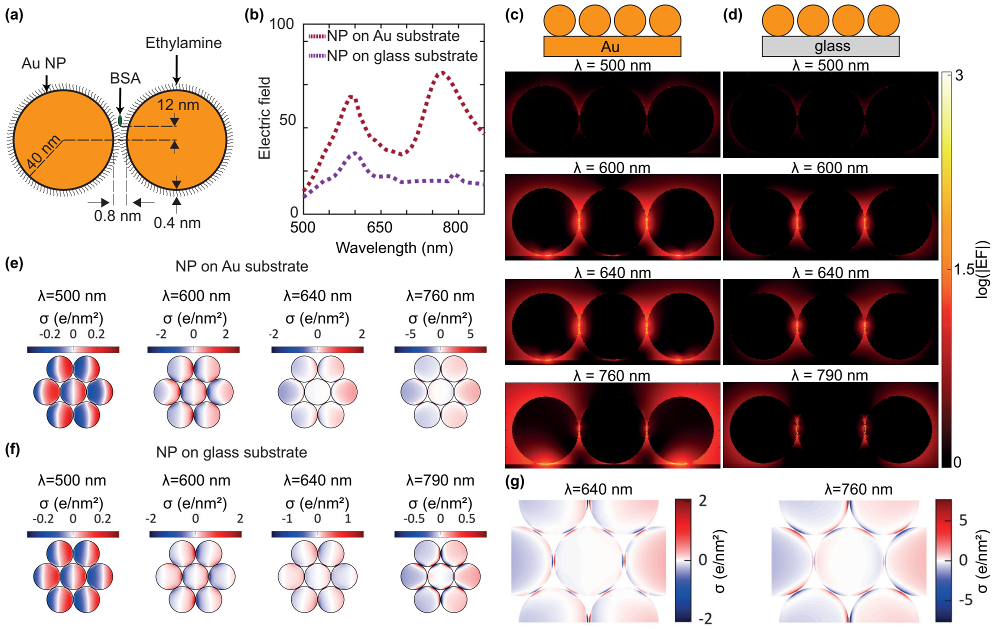

Supplementary Fig. 2 Simulated field distributions and charge maps of the plasmonic metasurface. (a) Schematic of the nanoparticle geometry showing 80 nm Au nanoparticles coated with an ethylamine shell and separated by 0.8 nm, with BSA dimensions ($4 \times 4 \times 14$ nm) indicated. **(b)** Simulated electric field enhancement as a function of excitation wavelength, showing strong resonance peaks in the visible–near-infrared region. **(c,d)** Electric field distributions ($\log|\mathrm{EF}|$) at different excitation wavelengths in the XY-plane for nanoparticle arrays on an Au substrate (**c**) and a glass substrate (**d**). The Au substrate supports strong nanocavity coupling and extended hotspot regions, whereas the glass substrate produces only weak confinement. **(e,f)** Charge distribution maps for Au nanoparticle on Au (**e**) and glass (**f**) substrates, illustrating the origin of the field confinement at different wavelengths. Strong charge localization is observed at nanoparticle junctions and nanoparticle–film gaps, confirming that interparticle coupling and substrate effects govern hotspot formation and their spectral response. **(g)** Zoomed-in charge distribution map of Au nanoparticles on Au substrate, showing that the charges are highly concentrated within the narrow interparticle gap region.

essential for achieving strong field confinement and enhancement near the metasurface. Each nanoparticle couples to its induced image charge in the underlying Au film, forming a nanocavity plasmon mode. These nanocavities further couple to one another, giving rise to collective plasmonic modes that extend the optical field farther from the surface compared to the nanoparticle-on-glass metasurface (Supplementary Figs. 2c,d). Such collective coupling increases the spatial reach and uniformity of the hotspot region, effectively broadening the volume in which proteins can interact with the enhanced electromagnetic field. The estimated accessible volume of a single 3D plasmonic hotspot, confined by three adjacent nanoparticles, is approximately 4.5×10^4 nm^3—nearly 200 times greater than the molecular volume of a BSA molecule. This accessible hotspot volume ensures that diffusing BSA molecules can readily enter the enhanced near-field region and experience sufficient electromagnetic coupling for single-molecule Raman detection without requiring immobilization.

Charge distribution calculations further highlight the physical origin of field enhancement (Supplementary Figs. 2e-g). At shorter wavelengths (500 nm), the charges are distributed on the entire surface of the nanoparticle, while at longer wavelengths (600 nm- 640 nm), the charges are localized at the edges of adjacent nanoparticles, resulting in a confinement of the near field. At 640 nm, coupling between nanoparticle–nanoparticle junctions and nanoparticle–film junctions results in hybridized plasmon modes that radiate more efficiently into the surrounding medium. At longer wavelengths (760–790 nm), the charge patterns become more strongly localized in the nanoparticle–nanoparticle junctions, leading to extended, strong near-field distributions. These results confirm that the hotspots in the array are governed by both interparticle spacing and nanoparticle-film coupling.

Overall, these findings demonstrate that the geometry of the Au NP array, combined with nanoparticle–film interactions, creates accessible plasmonic hotspots that are compatible with the size of single BSA molecules. This ensures that freely diffusing proteins can reproducibly access the regions of maximum field enhancement, which is essential for achieving robust single-molecule Raman detection.

SI. B Estimation of the number of molecules

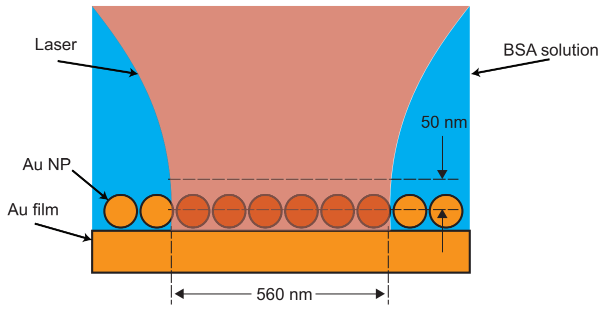

Supplementary Fig. 3 Measurement schematic of BSA solution on the plasmonic metasurface illuminated using a 1.4 NA objective.

We study single diffusing protein on the plasmonic metasurface using a 20 nM (concentration (ρ) can be written as, $\rho = 20 \times 10^{-9}$mol/L $= 1.20 \times 10^{16}$molecules/L) BSA solution, a concentration chosen to ensure single-molecule sensitivity under our experimental conditions. At this concentration, the average intermolecular spacing in solution is sufficiently large to ensure that, at any given time, only one BSA molecule occupies the enhanced near-field volume of a single hotspot. Simulation results show that the plasmonic field is confined within approximately $h = 50$ nm in the vertical direction, while extending laterally across the nanoparticle array, mostly between the particles. Considering the high numerical aperture (NA = 1.4) objective and the excitation wavelength used in the experiment, the focal spot diameter is estimated to be approximately $2r_s = 1.22\lambda/\text{NA} = 560$ nm [1]. Therefore the effective illuminated volume is given by $V_{ef} = \pi r_s^2 h = 1.23 \times 10^7 \text{nm}^3 = 1.23 \times 10^{-17}$L. Based on the

effective illuminated volume, we estimate an average occupancy of about $N = \rho V_{ef} = 1.20 \times 10^{16} \times 1.23 \times 10^{-17} = 0.14$ BSA molecule within this region, confirming that the detected Raman bursts arise from individual protein events.

Within this confined volume, diffusing BSA molecules occasionally enter and exit regions of high electromagnetic enhancement, producing discrete Raman scattering bursts that correspond to individual protein events. On average, only about 3% of the measurement trajectories exhibit detectable protein signals. The localized nature of the field ensures that these signals arise from single BSA molecules rather than ensemble contributions.

The combination of strong field confinement (within ~50 nm in z) and low protein concentration enables real-time detection of conformational transitions at the single-molecule level. These rare, transient events reflect spontaneous interactions of freely diffusing proteins with the plasmonic surface, demonstrating that our metasurface design provides both sufficient enhancement and spatial selectivity to resolve individual molecular dynamics.

SI. C Optical setup

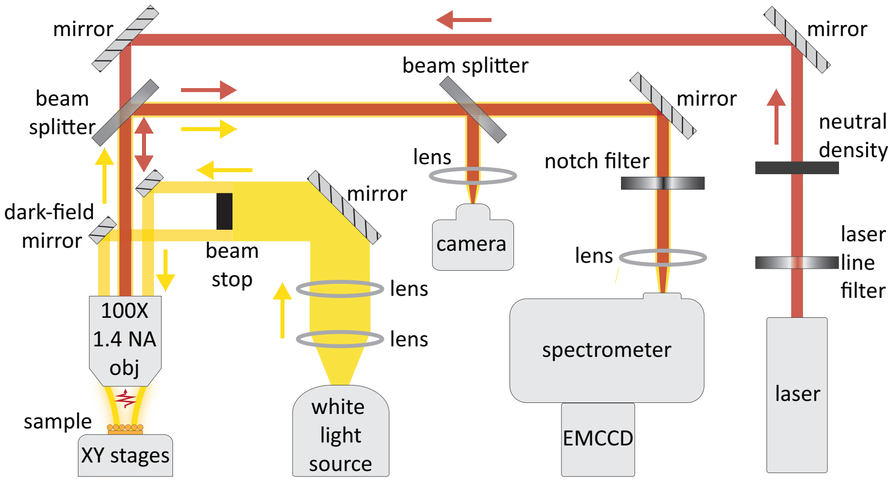

Supplementary Fig. 4 Schematic of the custom-made microspectrometer used for dark-field and SERS spectroscopy.

A custom-made microspectrometer setup used for single-molecule surface-enhanced Raman spectroscopy (SERS) and dark-field spectroscopy (Supplementary Fig. 4). A continuous-wave 640 nm laser (Cobolt Bolero 0640-05-01) serves as the excitation source, with a laser line filter (Thorlabs, FBH640-10) and neutral density filters (Thorlabs, NDC-25C-2M) controlling spectral purity and power, respectively. The beam is directed through a series of mirrors and coupled into the microscope via a beam splitter (Thorlabs, BSW26). A 100× oil-immersion objective lens (Olympus 100x/1.4 NA MPlanFL) focuses the laser onto the sample. The objective is mounted

on a z-piezo stage (Piezoconcept, FOC) to find the focal plane. The sample is mounted on a xy piezo stage (Piezoconcept, LFHS2) for positioning and scanning the sample. The scattered light in the reflection direction is collected using the same objective and split into two paths for imaging and spectroscopy. On the imaging path, a lens (f = 200 mm) focuses the reflected light that the objective collects on a camera (Basler, acA1440-220uc). In the other path, a notch filter (Thorlabs, NF633-33) blocks the light with the same wavelength as the excitation one, and a lens (f = 50 mm) focuses the light at the entrance slit of a spectrograph (Andor, Shamrock 500i). The spectrograph features a 300 lines/mm grating, paired with an EMCCD camera (Andor, Newton 970) that measures a spectrum with a spectral range of approximately 160 nm and a wavelength resolution of 0.1 nm. On the dark-field path, a white light source (Thorlabs, OSL2BIR) is coupled into the same optical path using a custom-made perforated mirror (diameter 34 mm, with a hole diameter of 18 mm) for dark-field illumination. The white light passes through the outer annular region of the objective's back aperture, generating a high-angle beam that illuminates the sample at angles greater than 65°, enabling dark-field microscopy. Only scattered light from the sample is collected through the same objective and directed toward a camera and spectrograph for imaging and spectroscopy. This dual-mode setup enables both dark-field imaging/ spectroscopy as well as SERS measurement.

SI. D Assignment of secondary structures from Raman peaks

To assign secondary structures based on the Amide I region of the Raman spectra, we compiled reported wavenumber ranges from the literature (Supplementary Table 1). These assignments vary slightly across studies in the literature due to differences in measurement technique, protein environment, and spectral deconvolution methods. These reference ranges guided our classification of BSA conformations under varying pH and surface conditions.

Supplementary Table 1 Amide I band assignments (in cm^{-1}) for secondary structures of BSA from various literature sources.

**Random**	**α-helix**	**β-sheet**	**Turns**	**Reference**
1671–1682	1651–1663	1630–1639	1689–1691	[2]
1662–1668	1650–1660	1667–1673	-	[3]
1680–1690	1650–1658	1660–1670	1670–1680	[4]
1640–1650	1650–1655	1660–1670	1670–1680	[5]
1640–1651	1650–1657	1670–1690	1655–1675, 1680–1696	[6]
1648–1650	1652–1659	1663–1672	1689–1691	[7]
1670–1680	1650–1655	1660–1670	1670–1680	[8]
-	1651–1659	1632–1640	1667–1675, 1679–1687	[9]
1640-1650	1650-1660	1660-1670	1670-1680	This work

SI. E Transition event frequency

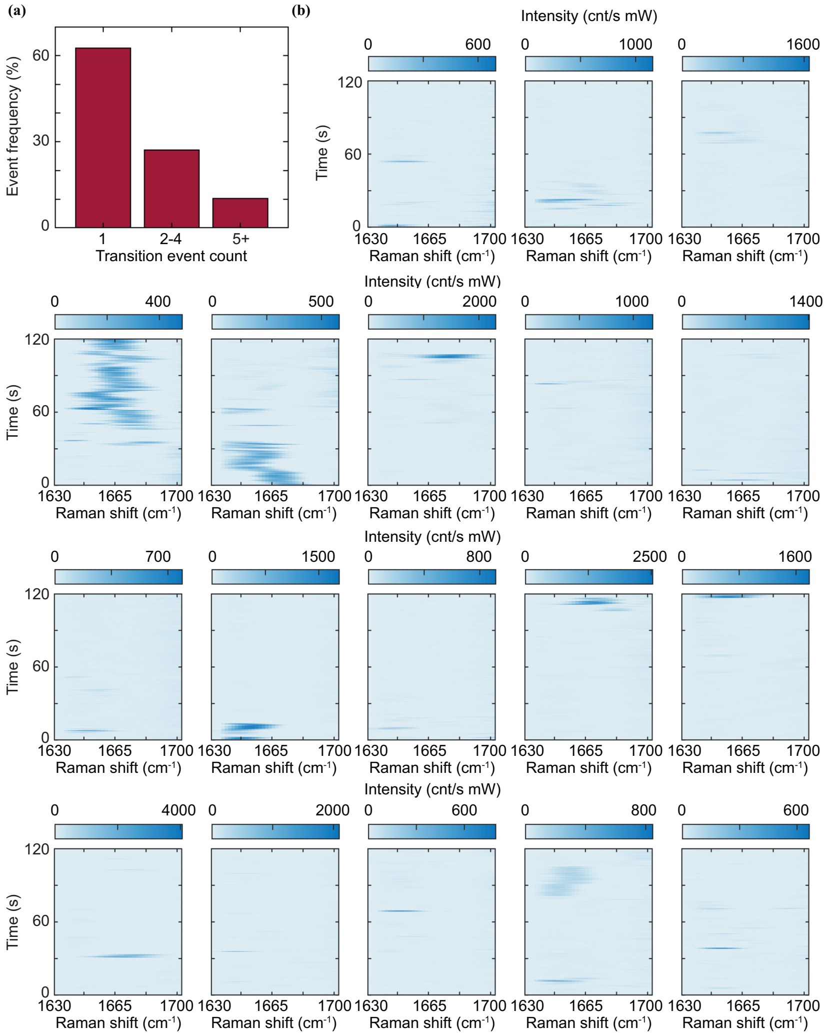

Supplementary Fig. 5 **(a)** Distribution of observed transition events per protein during single-molecule measurements. The majority of proteins exhibit only one detectable event, while a smaller fraction undergo multiple transitions (2–4 or 5+ events), indicating variability in dynamic behavior across the population. **(b)** Examples of time-resolved Raman maps of the Amide I band acquired show diverse interaction events.

To evaluate the dynamic behavior of individual BSA molecules, we quantified the number of transition events detected per protein trace. The majority of proteins (63%) exhibited only a single detectable event, while a smaller fraction showed multiple transitions (Supplementary Fig. 5a). Approximately 27% of proteins showed

2–4 events, and about 10% exhibited 5 or more. This distribution reflects the heterogeneous nature of protein dynamics at the single-molecule level, where most molecules interact briefly with the plasmonic surface, while a smaller subset remains in proximity for extended periods, allowing multiple detectable events. Representative time-resolved Raman traces illustrating single and multiple transition behaviors (Supplementary Fig. 5b), highlighting the diversity of conformational dynamics observed across individual protein events.

SI. F Data analysis procedure

To analyze time-resolved single-molecule Raman data, we developed a custom MATLAB workflow based on a modular class structure for spectroscopy acquisition and spectral analysis. The codes are available at https://github.com/MohammadRezaAghdaee/Spectroscopy. The complete analysis pipeline consists of the following key steps:

Data acquisition: Spectral data were acquired and stored in HDF5 format using the `Spectroscopy` class. This class interfaces with all hardware components, including cameras, shutters, objectives, and motorized stages, and coordinates the acquisition of both SERS and dark-field data under synchronized conditions.

Preprocessing: Acquired spectra were background-subtracted and normalized to the laser intensity and exposure time. The normalized spectra were cropped to the relevant Raman shift range (typically 1630-1700 cm^{-1}, covering the Amide I region). Conversion from wavelength to Raman shift was performed based on the excitation wavelength (639.6 nm). To reduce background contributions, reference spectra from water measurement in dynamic measurements and system baselines were subtracted from each measurement. Spectral time traces were extracted from the HDF5 files for each experimental condition. Metadata, including exposure time and acquisition parameters, were parsed automatically to align the spectra temporally and enable time-resolved analysis.

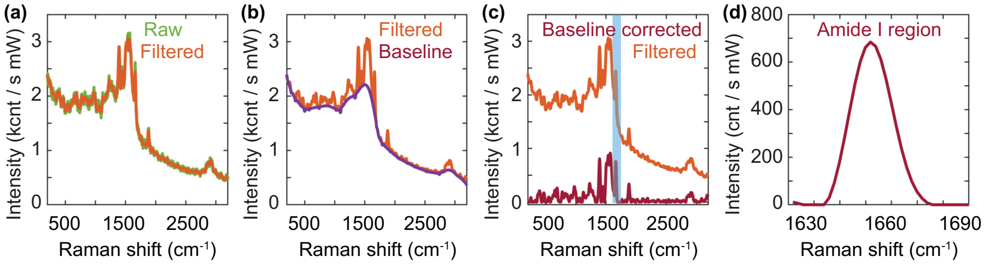

Supplementary Fig. 6 **(a)** Raw (green) and low-pass-filtered (orange) spectra showing suppression of high-frequency noise. **(b)** Baseline estimation (purple) applied to the filtered spectrum. **(c)** Baseline-corrected spectrum (red) revealing the Raman features of the protein; the highlighted area indicates the Amide I region. **(d)** Expanded view of the Amide I region corresponding to the secondary structure components of the protein.

Spectral analysis: Each time-resolved spectrum underwent baseline correction, Raman peak detection, and feature extraction using a custom-built analysis routine. The steps involved are as follows:

- Filtering: Raw spectra were smoothed using a low-pass filter (`lowpass` a MATLAB function) to reduce high-frequency noise while preserving peak sharpness (Supplementary Fig. 6a).
- Baseline correction: An adaptive baseline correction algorithm was applied. The method iteratively smooths the spectrum using variable window sizes and identifies the optimal baseline by minimizing the area between the original and stripped spectrum. This approach effectively removes broad emission without distorting the Raman features (Supplementary Fig. 6b, c).
- Automatic Raman peak detection: We performed a two-stage peak detection analysis. First, a coarse peak finding step was performed to identify local maxima that exceed predefined thresholds for intensity and prominence (using `findpeaks`, a MATLAB function). Next, we precisely identify the properties of the peaks by fitting a Gaussian function. The initial peak positions found from the coarse peak finding step serve as a guide for the initial fitting parameters of the Gaussian function. This, therefore, enables us to precisely extract the Raman peak position and intensity, enabling sub-pixel accuracy. The fitted Raman peak positions were mapped to corresponding secondary structures: α helix, β sheet, β turns, and random coil structures (Supplementary Figs. 6d, 7). This full analysis was performed frame-by-frame to preserve the temporal resolution of protein conformational dynamics.

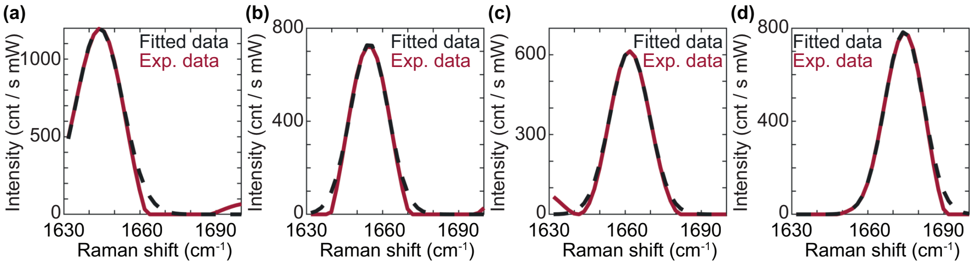

Supplementary Fig. 7 Examples of Raman peak detections at characteristic wavenumbers corresponding to **(a)** random coil, **(b)** α helix, **(c)** β sheet, and **(d)** β turn structures. The red spectra represent the experimental data, while the dashed black curves show the corresponding Gaussian fits.

Time dynamics: To investigate interaction kinetics, we tracked the evolution of Raman intensity in the Amide I band over time. Spectral 'on' states were defined as frames exhibiting resolved Raman peaks, while 'off' states lacked such features. Histograms of residence times in each state were generated, and a biexponential decay model was fitted to extract fast and slow dissociation rate components, reflecting transient and stable protein-surface interactions.

Histogram analysis: To quantify the distribution of structural conformations, Raman shift histograms were generated from the Gaussian-fitted peak positions in

the Amide I region. A bin width of 2.8 cm^{-1} was used to count occurrences of peaks assigned to specific secondary structures. These histograms reveal preferred conformational states and were generated for all detected events (Section SI.G).

Principal component analysis (PCA): Secondary structure classification (random coil, α helix, β sheet, β turn) was performed by mapping the Gaussian-fitted Raman peak positions to known Amide I assignments. All spectra (baseline-corrected spectra) were normalized to laser power and exposure time, and only spectra with protein events were retained. PCA was applied to this dataset to visualize clustering by structural class. The first two principal components (PC1 and PC2) were plotted with group-specific markers and colors, highlighting separation between conformations. This PCA visualization was complemented by bar plots of secondary structure proportions and a transition network diagram, showing the relative probabilities of interconversion between structural states.

SI. G Histogram of Raman peaks for different pHs

We analyzed the Raman spectra of BSA under neutral (pH 7), acidic (pH 3), and basic (pH 10) conditions and plotted the distributions of Raman peak positions as histograms. At pH 3, the Raman peaks shift toward longer wavenumbers, whereas at pH 10 they shift toward shorter wavenumbers relative to the neutral condition (Figs. 8b,c). These spectral changes indicate an increased presence of β sheet and random coil conformations, accompanied by a reduction in the α helical content. Consistently, PCA analysis shows a higher occurrence of β sheet and random structures under acidic and basic conditions compared to pH 7 (Figs. 3b,c). These results support the observations discussed in the main text, confirming that deviations from neutral pH promote partial unfolding and structural disorder in BSA.

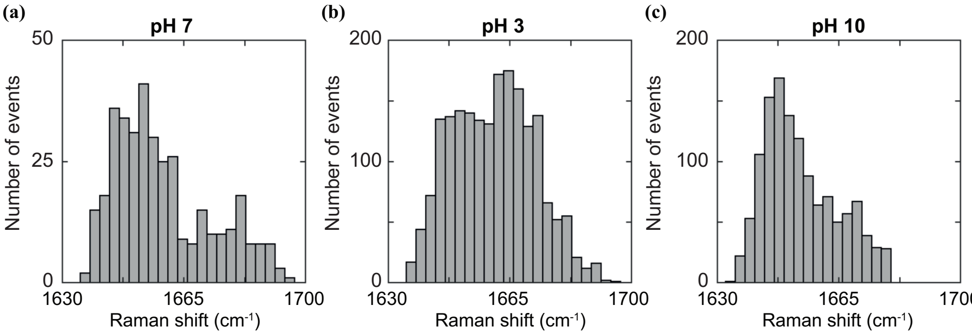

Supplementary Fig. 8 (a-c) Histograms of Raman shifts in the Amide I region, indicating the distribution of observed spectral features at pH 7, 3 and 10;

SI. H Charge parameters for protein–surface electrostatics

We summarize the effective net charge of bovine serum albumin (BSA) and the charge states of the functional groups at the pH values used. Charge fractions for carboxyl and amine groups were calculated using the Henderson-Hasselbalch relation [10] with solution pK_a values; BSA charges correspond to experimentally measured effective net charges.

Supplementary Table 2 Net charge of BSA and charge per surface functional group at different pH values.

Species	pH 3	pH 7	pH 10	Ref.
BSA (net charge, e)	+17	−8	−16	[11]
Methyl group (non-ionizable, e/site)	0	0	0	[12]
Carboxyl group ($pK_a \sim 4.7$, e/site)	−0.017	−0.994	−1.000	[13]
Amine group ($pK_a \sim 10.75$, e/site)	+1.00	+0.997	+0.15	[14]

SI. I Secondary structure of adsorbed proteins

To understand the influence of surface charge, we showed the secondary structure of BSA molecules adsorbed on the plasmonic metasurfaces in the main text. The metasurfaces were functionalized with cysteamine to expose an amine-terminated surface, ethylamine to provide a methyl-terminated surface, or with 3-mercaptopropionic acid to generate a carboxylate-terminated surface. After functionalization, the substrates were immersed in a 1 mM BSA solution for 24 hours to ensure sufficient adsorption and surface binding. We perform two-dimensional Raman scans across the substrate surface, and extract the peak intensities and positions from the acquired spectra (Supplementary Figs. 9a,b). These measurements reflect ensemble-averaged properties of immobilized protein populations, in contrast to the single-molecule analyses discussed in the main text.

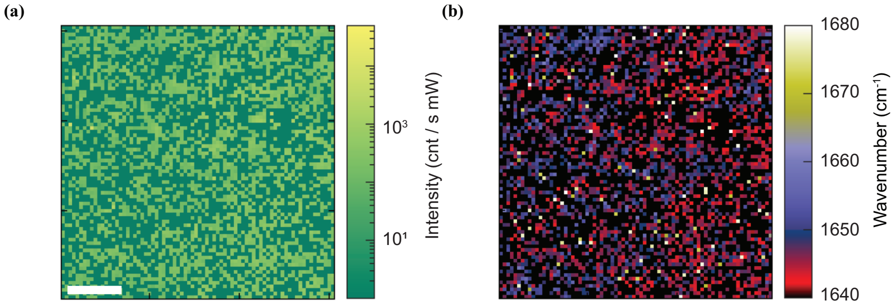

Supplementary Fig. 9 The 2D intensity map **(a)** and wavenumber map **(b)** obtained from the Raman spectra acquired from the metasurface with fixed BSA. The scale bar is 3 μm.

SI. J Concentration dependence of protein secondary structure

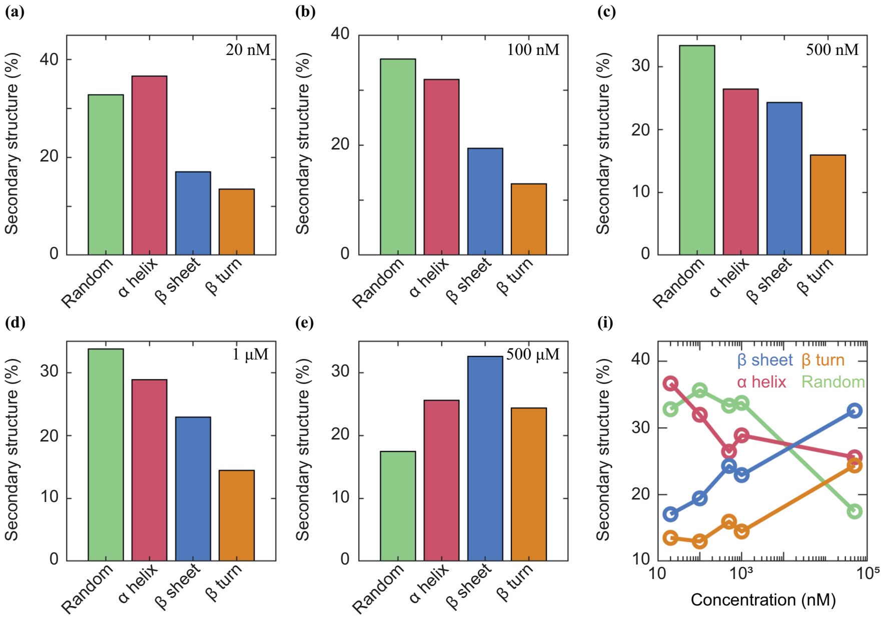

Supplementary Fig. 10 Concentration-dependent secondary structure distribution of single BSA molecules. **(a-e)** Bar plots showing the relative proportions of random coil, α helix, β sheet, and β turn conformations at 20 nM, 100 nM, 500 nM, 1 μM, and 500 μM BSA, obtained from time-resolved single-molecule SERS measurements. **(i)** Line plot summarizing the concentration dependence.

At low concentrations (20 nM and 100 nM), BSA molecules predominantly adopt α helical and random coil structures, while β sheet and β turn contents remain relatively low (Supplementary Fig. 10). As the concentration increases to the micromolar range, there is a clear reduction in α helix and random coil content, accompanied by a marked rise in β sheet structures, which become dominant at 500 μM. This concentration-dependent shift suggests that molecular crowding promotes β sheet formation, likely due to increased intermolecular interactions and aggregation-prone conformations.

SI. K Protein conformation free energy landscape

To obtain the free-energy landscape, we determine the state populations at temperature T using [15]:

$$p_i = \frac{N_i}{\sum_{j=1}^{n} N_j}, \tag{1}$$

where N_i is the number of observations assigned to conformation i. Relative free energies were then obtained by Boltzmann inversion [16, 17]:

$$G_i = -k_\mathrm{B} T \ln p_i + C, \tag{2}$$

with k_B the Boltzmann constant. The absolute offset C is arbitrary and was set to zero by subtracting the minimum value, $E_i = G_i - \min_j G_j$. These relative free energies were used to construct the protein–conformation free–energy landscape. The barrier height between adjacent conformations was defined as a scaled sum of their relative free energies. To visualize the landscape, the discrete relative free energy points were connected using a shape–preserving cubic Hermite spline, producing a smooth curve between conformations.

SI. L Fabrication of the plasmonic metasurface

To fabricate the plasmonic metasurface, Au NPs are first functionalized with ethylamine to promote self-assembly (Supplementary Fig. 11). A biphasic system is then prepared by adding hexane on top of the aqueous Au NP solution. Upon introducing acetone, the nanoparticles migrate to and self-assemble at the water–hexane interface, forming a uniform monolayer. This interfacial trapping is driven by a combination of ligand-mediated interactions and solvent polarity changes. The assembled monolayer is then transferred onto a template-stripped (TS) Au film. This process results in a well-ordered 2D array of Au NPs on a flat Au substrate, forming closely coupled nanocavities that serve as the plasmonic metasurface used in our experiments. We functionalize the plasmonic metasurface with these molecules such that the respective functional groups is exposed to the protein. Cysteamine binds to Au via its thiol and presents a protonated amine, yielding a positively charged surface; 3-mercaptopropionic acid also binds through its thiol group, leaving deprotonated carboxylate to expose a negative surface; and ethylamine yields a neutral, weakly interacting and nonpolar surface dominated by the methyl functional group.

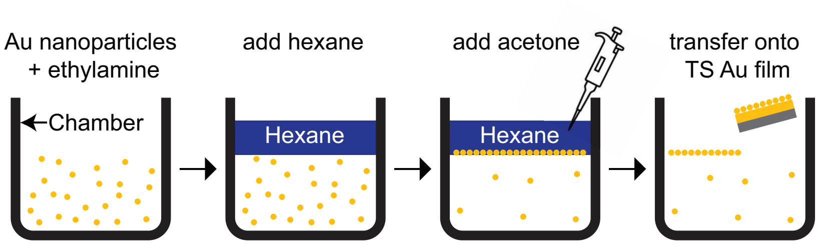

Supplementary Fig. 11 Schematic illustration of the interfacial self-assembly and transfer process for fabricating a 2D Au nanoparticle array on a template-stripped gold (TS Au) film. Ethylamine-capped Au nanoparticles are introduced into an aqueous chamber, followed by the addition of hexane to form a biphasic interface. Injection of acetone induces nanoparticle migration to the interface, where a monolayer forms. The assembled film is subsequently transferred onto a TS Au substrate, creating a coupled metasurface suitable for enhanced Raman spectroscopy.

References

[1] Murphy, D. B. & Davidson, M. W. *Fundamentals of light microscopy and electronic imaging* (John Wiley & Sons, 2012).

[2] Synytsya, A. & et al. J. raman spectrosc. *Journal of Raman Spectroscopy* **38**, 1646–1655 (2007).

[3] Wang, C.-H. & et al. Journal of raman spectroscopy. *Journal of Raman Spectroscopy* **47**, 940–947 (2016).

[4] Kuhar, N. & Umapathy, S. Single-molecule protein conformation analysis. *Analytical Chemistry* **92**, 13509–13517 (2020).

[5] Kuhar, N., Sil, S. & Umapathy, S. Protein structure from raman spectroscopy. *Spectrochimica Acta Part A: Molecular and Biomolecular Spectroscopy* **258**, 119712 (2021).

[6] Pelton, J. T. & McLean, L. R. Spectroscopic methods for protein analysis. *Analytical Biochemistry* **277**, 167–176 (2000).

[7] Sane, S. U. & et al. Bsa structure studies. *Analytical Biochemistry* **269**, 255–272 (1999).

[8] Kuhar, N. & et al. Nanoparticle-enhanced protein analysis. *RSC Advances* **8**, 25888–25908 (2018).

[9] Fedotova, E. & Paston, S. Protein spectroscopy by amide i band analysis. *St. Petersburg State Polytechnical University Journal. Physics and Mathematics* **17**, 331–335 (2024).

[10] Henderson, L. J. Concerning the relationship between the strength of acids and their capacity to preserve neutrality. *American Journal of Physiology-Legacy Content* **21**, 173–179 (1908).

[11] Böhme, U. & Scheler, U. Effective charge of bovine serum albumin determined by electrophoresis nmr. *Chemical Physics Letters* **435**, 342–345 (2007).

[12] Israelachvili, J. N. *Intermolecular and surface forces* (Academic press, 2011).

[13] Wagner, F. S. & Staff, U. b. Acetic acid. *Kirk-Othmer Encyclopedia of Chemical Technology* 1–21 (2000).

[14] Atallah, C., Charcosset, C. & Greige-Gerges, H. Challenges for cysteamine stabilization, quantification, and biological effects improvement. *Journal of Pharmaceutical Analysis* **10**, 499–516 (2020).

[15] Bryan, J. S. & Pressé, S. Learning continuous potentials from smfret. *Biophysical journal* **122**, 433–441 (2023).

[16] Ramanathan, R. & Munoz, V. A method for extracting the free energy surface and conformational dynamics of fast-folding proteins from single molecule photon trajectories. *The Journal of Physical Chemistry B* **119**, 7944–7956 (2015).

[17] Covino, R., Woodside, M. T., Hummer, G., Szabo, A. & Cossio, P. Molecular free energy profiles from force spectroscopy experiments by inversion of observed committors. *The Journal of chemical physics* **151** (2019).
